# The Treatment of Liver Metastases in Patients with Neuroendocrine Tumors in 2012

**DOI:** 10.1155/2013/702167

**Published:** 2013-02-14

**Authors:** Daniela Macedo, Teresa Amaral, Isabel Fernandes, Ana Rita Sousa, Ana Lúcia Costa, Isabel Távora, António Quintela, Paulo Cortes, Luís Costa

**Affiliations:** ^1^Oncology Division, Hospital de Santa Maria, Lisbon, Portugal; ^2^Instituto de Medicina Molecular, University of Lisbon, Portugal; ^3^Radiology Division, Hospital de Santa Maria, Lisbon AV Professor Egas Moniz, 1649-039 Lisbon, Portugal

## Abstract

Neuroendocrine tumors (NETs) comprise a heterogeneous group of tumors that form a distinct entity. Approximately 75–80% of patients present with liver metastases at the time of their diagnosis, and 20%–25% will develop these lesions in the course of their disease. The presence of secondary deposits in the liver significantly increases the morbidity and mortality in these patients. The only potentially curative treatment is the surgical resection of the primary tumor and hepatic lesions. However, only 10% of patients presents under ideal conditions for that approach. Several techniques aimed at localized liver lesions have been applied also with interesting results in terms of survival and symptom control. The same has been demonstrated with new systemic therapies (target therapies). However, these are still under study, in order to define their true role in the management of these patients. This paper intends to address, in a general way, the various treatment options in patients with liver metastases from neuroendocrine tumors.

## 1. Introduction 

The neuroendocrine tumors (NETs) are a heterogeneous group of neoplasms that share some biological characteristics, that have been addressed as a common entity [1]. In 62%–82% of the cases, the NETs are located in the digestive system, being designated as gastroenteropancreatic neuroendocrine tumors (GEP-NETs) [2].

The latest figures from the UK, Sweden, and Switzerland suggested that their incidence is 2-3/100000 with a higher prevalence in females [1]. Also data from the Surveillance, Epidemiology, and End Results (SEER) Program Database report an increased incidence between 1973 and 2004 of 1.09 to 5,25/100000 [3, 4]. 

NETs are classically characterized by their ability to secrete hormones and/or vasoactive peptides, which results in many of their clinical manifestations, ganging up on specific hormonal syndromes. The most frequent syndrome is the carcinoid syndrome, which results from the secretion of serotonin and includes symptoms such as skin flushing, severe diarrhea, abdominal cramping, and electrolyte abnormalities [4, 5]. Their clinical course is variable, having been considered traditionally with an indolent natural history [1, 4]. However, some NETs have aggressive behavior regarding the rapid progression of metastatic lesions. All of them have the potential of metastatic dissemination and, therefore, are currently recognized as malignant neoplasms [6]. The prognosis and the approach of NETs are guided mainly according to the histological classification. The tumors with grade 3, mitotic count exceeding 20/10 high-powered fields and/or with Ki-67 proliferative index exceeding 20% represent aggressive malignant disease with rapid clinical course and a low survival [4].

The liver is the most important place of metastatic disease [7]. More than 75% of patients with NETs of the small intestine and 30%–85% of patients with pancreatic NETs have liver metastases at the time of diagnosis or during the course of their disease. Additionally 5%–10% of patients with NETs of unknown primary location have liver metastases at the time of the diagnosis [6, 8].

Patients with liver metastases have a worsening of their morbidity and mortality, as compared to patients without these lesions. According to Touzios et al. [9] survival rate to 5 years of patients with untreated liver metastasis is about 13%–54%, against 75%–99% in patients without liver metastases [6, 9]. This revision aims to address the theme of GEP-NETs with metastatic liver, their clinical significance, prognosis, and its current therapeutic approach with a special focus on medical treatment.

## 2. The Clinical Significance and Prognosis of Liver Metastases 

Patients with NETs often develop liver metastases (NLMs) affecting significantly their morbidity and mortality. About 75%–80% of patients present with hepatic metastasis at the time of diagnosis (synchronous), while 20%–25% develop secondary lesions in the course of their illness (metachronous) [10]. The 5-year survival rate for patients with NLMs with support therapy is 0%–20% [11].

The presence of liver metastases is closely related to the appearance/aggravation of symptoms. Many peptides and hormones are metabolized in liver. The presence of hepatic metastases leads to less metabolization of these peptides leading to an increase of their circulating levels. Sometimes, only then, the complete disease phenotype is present. Consequently, the symptomatic control is an essential aspect of the treatment of NLMs [5].

Hepatic involvement is an important prognostic factor, despite the primary site of tumor. Its distribution, in the way it modifies therapeutic management, reflects the aggressiveness of the tumor. Liver metastases are divided into 3 types according to the localization. Type I metastases are lesions only confined to one hepatic lobe or limited to two adjacent segments, corresponding to about 20%–25% of cases. Type II metastases include the presence of dominant injury with small satellites contralateral (10%–15% of cases). Type III metastases correspond to diffuse multifocal, being the most prevalent being (60%–70% cases). Because of the possibility of surgical resection, type I is associated with a favorable outcome. Type III metastases have a worse prognosis regarding the greater liver involvement and the lack of surgical approach. In this group of patients, liver transplantation can be considered a form of treatment [6].

## 3. Liver-Targeted Therapies 

Surgery consistently proved its superiority in relation to nonsurgical therapies. However, the complete excision of secondary lesions is only an option for a small number of patients (10%–20%) [6].

Therapeutic options for patients without surgical indication have been improved in the latest years [12]. It is known that the liver metastases are usually highly vascular lesions. Vascularization is originated in the hepatic artery in more than 90% of the cases. This differs from the normal vascularization system which is derived from the venous system port [4]. The handling of hepatic artery prevents the supply of nutrients and oxygen to the tumor cells with its consequent destruction. For that reason, several techniques have been developed by exploiting duality in the liver vasculature in order to control the disease process [4]. These modalities are include the ablative techniques, the hepatic artery embolization, chemoembolization, and radioembolization (selective internal radiation therapy) [12]. Patients with predominant metastatic liver disease are those who benefit the most from these therapies [13].

### 3.1. Surgical Treatment

The surgical resection is the only potentially curative treatment in NETs. Curative intention depends on the stage and presentation of the disease [13, 14]. Even with hepatic involvement, surgical approach, when feasible, is the best treatment option, with proven benefits in terms of overall survival and quality of life improvement [1, 6, 12]. Patients treated with surgical resection of the primary tumor and liver metastases had a survival at 5 years of more than 60%, reaching 80% in some studies, with minimum mortality (less than 5%) and admissible morbidity (less than 30%) [6, 12]. Patients with liver metastasis that are not candidates to surgical treatment (80%–90% of cases), still benefit from primary tumor resection. This approach can be combined with other liver-direct therapies increasing patients' outcome [12, 13, 15]. In these cases, where primary tumor was resected, a better symptomatic control was achieved in about 90% of the patients. Therefore, the palliation of the symptoms is also a criterion for surgery [5, 6]. 

Regarding the criteria for selecting patients with NLMs, there is no consensus, since the several studies developed included a small number of patients and different surgical procedures. However, when the complete excision of a significant number of liver metastasis is possible, surgery must always be considered. In a recent retrospective study, 74 cases were analyzed and the 5-year survival rate was more than 60% in all patients who underwent surgical resection of liver metastases. In 65% of these patients, excision was incomplete, and there was no worsening of the prognosis [6, 12]. However, the identification of other predictive variables in patients' selection for surgery was needed. Therefore, clinical studies were conducted, and patients were selected based on tumor grade. The analyses of the results from these studies showed that tumors with high grade had a worse postsurgical outcome when compared to low grade tumors [12]. High grade NET tumors are good candidates to chemotherapy and do not seem to beneficiate from surgery.

Translating these results into clinical practice, in 2008, the European Neuroendocrine Tumor Society (ENETS) issued general guidelines for surgical resection based on the three types of liver involvement. For patients with hepatic involvement of type I surgical resection is the first therapeutic option, while in patients with the involvement of type III it is totally excluded [6]. In order to give the best possible treatment to patients with NET tumors, a careful evaluation should be performed prior to surgery. This evaluation should take into account the following aspects: performance status, localization of the primary tumor, tumor grade, possibility of primary tumor resection, presence and localization of metastases, possibility of complete resection of metastases, presence of extrahepatic disease (extrahepatic disease should be excluded based on adequate image methods), and the presence or absence of carcinoid heart disease [6, 17]. In patients with carcinoid heart disease, symptoms control and clinical stabilization should be achieved before aggressive surgery is considered. Also, in patients with carcinoid syndrome, even in those under treatment with somatostatin analogues, intra- and postoperative carcinoid crisis must be prevented, using these same somatostatin analogues [6]. Octreotide should be readily available during any surgical procedure. Preoperative administration of octreotide (300 micrograms subcutaneously) can reduce the incidence of carcinoid crisis and is recommended for patients with a history of carcinoid syndrome who require surgical procedures. Treatment for carcinoid crisis differs from that for other causes of acute intraoperative hypotension. Symptoms are generally refractory to fluid resuscitation alone. Calcium and catecholamines may provoke the release of mediators from the tumor and worsen, rather than ameliorate, the syndrome. During a carcinoid crisis, the blood pressure should be supported by the infusion of plasma and octreotide (300 micrograms IV) given immediately. A continuous IV drip of octreotide may be needed. In patients with high risk of carcinoid crisis, an IV octreotide drip may be initiated preoperatively [1, 18–23]. 

The presence of abdominal lymph node involvement, including local recurrence, is not an absolute contraindication for surgery, if the excision of lymph nodes and liver metastases and/or recurrent lesions could be done simultaneously or in planned staggered phases [6].

Recurrence after resection is common with the 5-year recurrence rate being greater than 97%. Therefore, surgical resection should be as radical as possible. Extensive lymphadenectomy and a careful exploration of the entire abdominal cavity should be carried out during surgical procedure [24]. The use of intraoperative echography seems to be of additional value as it allows to define the extent of known lesions and to detect small additional lesions [6].

Cytoreductive surgery may be useful for a small and restricted group of patients. It is usually recommended in cases where more than 90% of the tumor can be excised or in young patients [6, 12, 20, 25, 26]. Symptomatic patients also benefit from cytoreductive surgery, as it seems to improve the effectiveness of medical therapy [12]. 

### 3.2. Liver Transplantation

Liver transplantation is a therapeutic approach with an intention to cure, to prolong survival or control of carcinoid symptoms. Liver transplantation is only indicated in cases of metastatic disease if the primary tumor is an NET [12]. In 1998, Lehnert analyzed a total of 103 NET patients with liver transplant. The overall 5-year survival was 47% with disease-free survival of 24%. The tumor histology and its primary location did not affect the survival in this study, unlike the extent of surgery and the patient's age [12, 27]. However, in a more recent clinical trial, primary location in the duodenum or pancreas was associated with worse prognosis after transplant [6, 12, 28]. These findings were, however, not supported by other investigations [12, 29].

Regarding biomarkers analyses and their correlation with transplant outcome, the authors found that patients with a low Ki-67 and normal expression of E-cadherin had a more favorable prognosis. On the other hand, p53 status had no influence on prognosis [2]. The analysis of the data from the United Network for Organ Sharing between November 1988 and March 2011 showed that only 185 liver transplants were due to NLMs. The 5-year overall survival was 57.8%, lower than patients transplanted for other reasons/with other pathologies [12, 14].

In patients with diffuse and unresectable liver disease, with uncontrolled symptoms (resistant to medical therapy support), liver transplantation should be considered and may be an option in carefully selected patients [12]. The main criteria for selecting patients for liver transplantation are in these cases being not candidate for resection, diagnosis, and complete resection of the primary tumor at least one year before, no extrahepatic disease, stable disease at least for one year, and failure of nonoperative treatments [12]. However, liver transplantation remains controversial.

### 3.3. Ablative Techniques: Radiofrequency Ablation

Patients with NET tumors can be selected to treatment with different types of ablative techniques such as cryoablation, alcohol ablation (these two are less used), and radiofrequency ablation (RFA). 

Cryoablation is the most studied thermoablation technique [30]. Decreasing temperature lowers cell viability, and this depends both on the rate of colling and the spatial relationship between the ice formation and the cryoablation probe [31]. The lower the temperature (which should be −50°C) the highest the chance of achieving tissue necrosis [32].

Alcohol ablation also known as percutaneous alcohol injection (PAI) has already been studied in different series [33, 34]. This technique could be of advantage in patients whose liver metastases are located next to large vessels or the bile duct, that could be damaged by the heat released during RFA [14]. These lesions have to be less than 5 cm [14]. PAI should be used not as monotherapy but with other ablative techniques in order to achieve better results [35].

RFA consists in intense heat production from radiofrequency waves through alternating electric current and can be performed by percutaneous or laparoscopic approach [12, 13]. Such phenomenon causes intracellular ion vibration leading to cell destruction [14]. It is currently the preferred ablation technique of most major centers. Its use has shown benefit in local disease control with rapid symptomatic relief [6]. It is mainly used in patients with a small number of liver lesions that cannot be resected or as an “adjuvant” to other therapies [12].

The largest clinical study until involving patients with hepatic lesions treated with RFA was reported by the Cleveland Clinic. Eighty-nine patients were followed and submitted to a total of 119 laparoscopic RFAs. Results showed that about 90% of the patients experienced immediate relief of symptoms after the procedure, with a mean progression-free survival of 1.3 years. From these 89 patients, 22% had recurrence, and from these 22% recurrences, 63% corresponded to new liver lesions and 59% to extrahepatic disease [36].

An important limitation of the effectiveness of this technique is the size of the tumor. It seems to be difficult to totally eradicate lesions greater than 3 cm, and lesions greater than 5 cm are unsuitable for RFA, as demonstrated by Mazzagalia et al. [1, 6, 12]. A maximum of five lesions is considered the ideal number for this therapy. The presence of a higher number of metastatic bilobar lesions contraindicates the procedure [17]. 

RFA is a procedure with low morbidity (5%–12%) and low 30-day mortality (0%-1%) [12, 17]. The most frequent complications include hepatic abscesses, carcinoid crisis, biliopleural fistula, bile leakage, and pleural effusions [12].

### 3.4. Hepatic Artery Embolization and Hepatic Artery Chemoembolization

Hepatic artery embolization (HAE) and hepatic artery chemoembolization (HACE) are two different technics that can also be used in the treatment of liver metastasis. Hepatic arterial embolization is frequently applied as a palliative technique in patients with hepatic-predominant metastatic NET who are not candidates for surgical resection. It is based on the principle that tumors in the liver derive most of their blood supply from the hepatic artery, whereas healthy hepatocytes derive most of their blood supply from the portal vein.

Using this technic, we can block blood supply with nutrients and oxygen to the tumor with consequent ischemia/necrosis [5]. This technique has also proven to achieve the reduction of lesions size as well as symptoms relive [1, 12]. Retrospective evaluation showed that a high rate and a prolonged disease regression were achieved with chemotherapy after HAE. Therefore, cytostatic agents were added to this technique resulting in hepatic artery chemoembolization (HACE). The most commonly used are doxorubicin, mitomycin C, streptozin, and cisplatin [24]. HACE provides not only the embolic blocking but also the supply of direct chemotherapy to these cells, increasing the action/concentration of these agents (more than 20 times of the systemic chemotherapy) [12]. Its benefits have been documented by different authors, but there were no comparative studies between HAE and HACE demonstrating superiority of one of these techniques [1].

Both HAE and HACE are palliative techniques used in patients with unresectable lesions, with diffuse and progressive liver disease, with symptoms not controlled with medical treatment and without impaired liver function [13, 24]. The main benefit is symptomatic relief (70%–90% of the patients) and tumor growth control (50% of the patients) [1, 24]. The mean duration of response may be short (up to allow collateral circulation to develop) ranging from 6–42 months in uncontrolled series of patients [5, 6]. 

These therapies have proved to be a valuable option, particularly in patients with more than 75% of hepatic involvement, carefully selected, without additional risk factors [12]. 

Just as in other therapeutic procedures might exist associated complications, sometimes severe. Sepsis, liver and renal failure, carcinoid crisis, necrotizing cholecystitis, and peptic ulcer bleeding have been reported in about 7.5 to 23.8% of patients. The most frequent is the postembolization syndrome (i.e., fever, abdominal pain, leukocytosis, increased transaminases, and bilirubin), which occurs in 90% of patients, in most cases self-limited (24–72 hours of duration) [6, 17, 24]. In order to minimize the complications, it is recommended to split the area to be treated in small portions in each session (one lobe per session) [5, 17]. Multiple sessions are usually required and must be carried out with 4–6 week interval [6, 13]. Concomitant administration of antibiotics and somatostatin analogues, as well as aggressive hydration, to prevent liver abscesses, carcinoid crisis, and tumor lysis syndrome is further recommended [1, 24]. The use of somatostatin analogues during the procedure is of extreme importance in order to prevent carcinoid crisis. The most relevant contraindications are bleeding disorders, renal failure, and/or hepatic vein occlusion [12]. 

### 3.5. Selective Internal Radiation Microsphere Therapy (SIRT)

External radiation therapy has limited value in NETs, being only indicated in the presence of symptomatic brain and bone metastases [37]. SIRT is a novel technique based on small microspheres radioisotopes, including Yttrium 90 (90Y), which are introduced directly into the hepatic artery, leading to embolic blocking of tumor cells and also exposing them to radioactive agents. 

Saxena et al. [38] investigated the safety and efficacy of this treatment in patients with NLMs. In this study, 34 patients had long-term responses with a mean overall survival of 29.4 months with radiological improvement in 50% of the patients. Cromogranina A levels decreased to 50%, with a maintained response for approximately 30 months [12, 38]. Kennedy et al. [39] also showed that the major benefit seems to stabilize advanced disease allowing an increase in overall survival although still under investigation results have been promising. 

Complications such as postembolization syndrome are common, with a small risk of radiation gastritis and ulceration [24]. 

More studies are needed to realize the best use of this treatment.

## 4. Systemic Therapy

Since NETs are a rare pathology, large randomized studies in patients with NLMs are difficult to perform. This limitation leads to an overall assessment of restricted systemic therapies that cannot be translated in to a consensual treatment algorithm. However, the data obtained from a single center study showed that the aggressive medical treatment of NLMs instead of surgical therapy increases the 3-year survival rate to 76.4% and the 5-year survival rate to 63.9%. Therefore, conservative treatment, that is, “wait and watch,” is no longer accepted [12].

### 4.1. Somatostatin Analogs (SSA)

The majority of NETs is expressed on the surface of their cells somatostatin receptors (SSTR 1–5). Their activation inhibits the secretion of peptides and amines by tumor cells and also the effect of tumor growth factors inducing apoptosis [12, 24]. Since natural somatostatin has a very short half life (2-3 min) analogues with longer half lives have been developed for clinical use [24]. In 1980, Bauer et al. [40] synthesized the first analogue-octreotide.

The main use of SSA is in symptomatic control. In an initial study, the subcutaneous administration of octreotide at the dose of 150 mg TID improved symptoms, especially carcinoid syndrome, in 88% of patients [39]. This drug has been used extensively in the last three decades. A long-acting formulation was developed that allows a single monthly administration. However, the short action formulations continue to play an important role in stabilizing the disease, especially in the situations of carcinoid crisis and its prevention [24].

In more recent years, there has been evidence that SSA can have antitumor activity and also be able to reduce tumor growth by direct action on somatostatin receptors. It is even possible that specific receptors can induce apoptosis [13]. The first randomized clinical study that demonstrates the possible anti-tumor effects of long-acting octreotide in comparison to placebo was the PROMID study [41]. This study showed a decreased risk of disease progression of 66% and arrested tumor growth in 69% for a median of 14.3 months [12]. Stable disease was often registered as the better response [41]. Treatment with LAR octreotide seems to be more effective in patients with low hepatic tumor burden and resected primary tumor [4, 41]. Scintigraphy with somatostatin allows, beyond the level of self-uptake, to predict therapy's patient response. There are other SSAs in use such as lanreotide and others in the study as pasireotide (this one can interact with four receptors (ssts1, ssts2, ssts3, and ssts5) while octreotide and lanreotide only interacted with two type of receptors) [42]. Preliminary data suggest that pasireotide could be useful in patients that do not respond to octreotide and could be able to control symptoms in 27% patients [43].

 Severe adverse events with SSA therapy are infrequent. However, nausea, vomiting, diarrhea, steatorrhea, cardiac abnormalities, arrhythmias, hypothyroidism, and hypoglycemia were reported in different percentages [42, 44, 45]. Cholecystitis can occur in more than 50% of cases due to its action as an inhibitor of the contractility of the gallbladder. In patients with risk factors, prior elective cholecystectomy may be necessary [46].

### 4.2. Interferon Alfa (INF-*α*)

Interferon is an immunomodulator that inhibits the production of several growth factors and also has antiangiogenic properties. When used in NET's treatment these agents seem to have an additional activity which is the hyperregulation of somatostatin receptors [13]. Interferon *α* may be used in functioning and nonfunctioning NETs, alone or in combination with SSA in view of the lack of response under maximum dose of these agents [1].

In some clinical studies, treatment with INF-*α* showed symptomatic control in 30%–70% of cases, with stable disease in more than 70% of patients [12, 47]. However, other studies have not demonstrated their effectiveness even in association with the SSA. A 37% increase in 5-year survival with INF-*α* alone to 57% in combination with octreotide was observed in a single study [48]. However, the results were not statistically significant due to the small number of patients. Two other small randomized studies showed that the combined therapy increased toxicity without additional gain in survival [49, 50]. Emerging evidence suggests that pegylated interferon is better tolerated and also shows some activity. However, investigations are still ongoing [1, 12].

### 4.3. Peptide Receptor Radionuclide Therapy (PRRT)

PRRT is a therapeutic option with particular advantages in patients with a symptomatic disease and tumors with positive somatostatin receptors, not candidates for surgery. This technique uses a radioactive peptide bound to SSA, which after the interaction with the receptor is internalized, releasing specific and localized radioactivity, allowing a precise destruction of tumor cells [42, 51, 52]. It has little interference with the nontumor tissue, except with the kidney, bladder, and bone marrow [52]. Many radionuclides have been developed. The most used are ^177^Lutetium and ^90^Y, which differ from each other in particles emission, particles energy, and tissue penetration [6, 12]. PRRT with ^177^Lu-DOTA^0^Tyr^3^ octreotate seems to be more effective. Tumor response rate of 35% and disease stabilization of 80%–90% can be achieved [52]. After treatment with these agents the mean duration of response is 30–36 months [53]. 

Scintigraphy with somatostatin can predict the efficacy of PRRT as with the SSA. Low uptake indicates 20% of the possibility of the effect on liver metastasis, and high uptake indicates the possibility of 60% effectiveness [53]. 

### 4.4. Chemotherapy

NETs sensitivity to cytotoxic therapy appears to have correlation with the primary location of the tumor and tumor grade [13]. The specificity of cytostatic in NLMs is not evident. Clinical studies show their use in NETs in general, without specifications for metastatic liver disease. The main studies appeared in pancreatic NETs [12]. The pancreatic NETs have been successfully treated with streptozocin (STZ). The great effectiveness of their use is in combination with other agents such as 5-fluorouracil and doxorubicin, but with results of mean response of 9.3 months [12, 13].

Dacarbazine is another cytostatic agent with proof of effectiveness in pancreatic NETs, with a response rate of 34% in phase II study [54]. Clinical use of dacarbazine with STZ has had limitations due to its high toxicity [13]. Recently, the use of alkylating agents such as temozolomide appears to be promising in this tumor type. In a phase II study thalidomide and temozolamide demonstrated a response rate of 45% [55]. In a retrospective study with temozolamide and capecitabine, the response rate was 70%, with a mean progression-free survival of 18 months and 2 years, overall survival of 92% [56].

Platinum based chemotherapy may be useful in patients with high grade undifferentiated tumors and with liver metastases. In these patients, chemotherapy is the first line treatment. Response rate was 42%–80% using cisplatin and etoposide and 78% with combination of oxaliplatin [57–60].

Although chemotherapy could be used as rescue treatment, it is not considered the first line of the nonsurgical treatment. Furthermore, the presence of liver metastases is associated with a poor response compared to NETs without liver metastasis [12]. 

### 4.5. Target Therapies

Conventional chemotherapeutic agents have limited efficacy in metastasized NETs. Despite positive results in some way to the little differentiated carcinomas and primary for the pancreas in relation to other locations, overall response rates are low and the impact on survival is small [1]. The growing knowledge about the biology of these tumors, along with the ability to synthesize new drugs that interfere with therapeutic targets, has given the possibility to develop new target therapies that can change the outcome of these patients. 

NETs are highly vascular tumors that express on their cell surface vascular endothelial growth factor (VEGF) receptors. Tumor progression seems to be associated with high levels of circulating VEGF, making this possible therapeutic target, such as in other neoplasms [1].

Bevacizumab (a monoclonal antibody), sunitinib, sorafenib, and pazopanib (tyrosine kinase inhibitors) have been the most studied drugs in pancreatic NETs [6].

Bevacizumab was introduced in a randomized clinical study with patients being treated with SSA and bevacizumab compared to the combined therapy with Peg INF-*α*. Superiority was seen in bevacizumab treatment arm with 95% of patients with a progression-free survival of 18 months [61].

**Figure 1:**
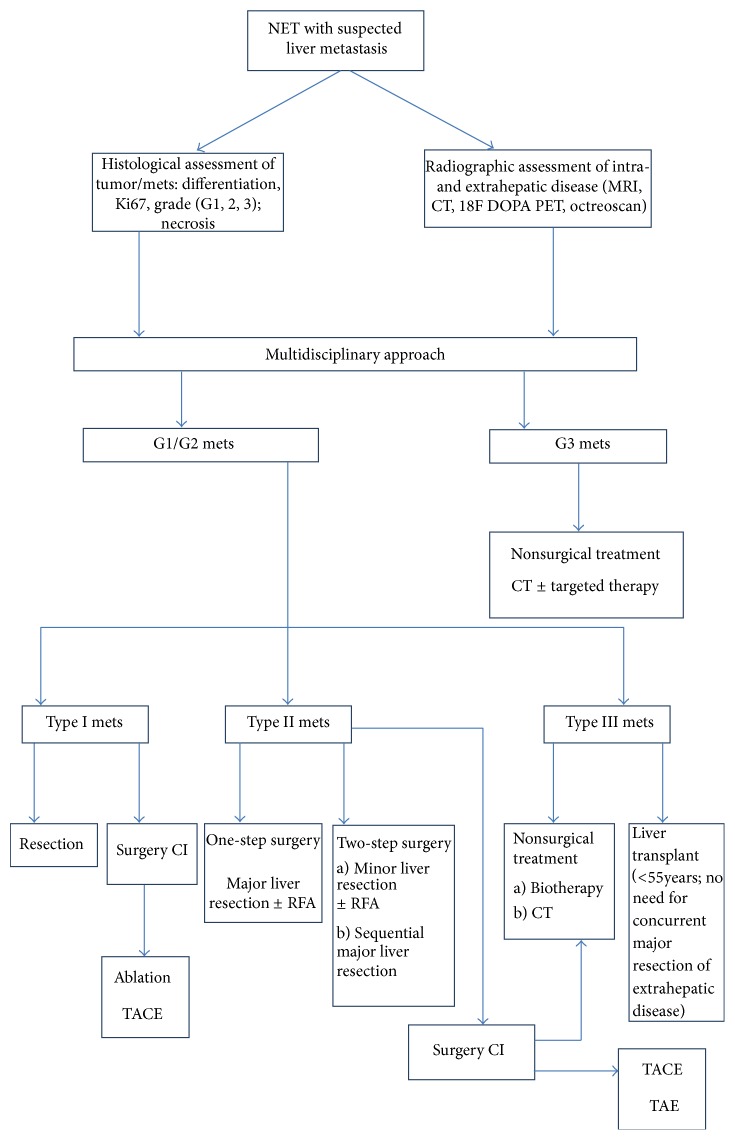
Algorithm treatment in patients with neuroendocrine tumors and hepatic metastases (adapted from [12, 14, 16]).

Sunitinib has been the most studied agent. Based on positive results from phase II studies, sunitinib was tested in a double-blind phase III study in patients with well-differentiated and progressive pancreatic NET compared with placebo. The primary endpoint (progression-free survival) was statistically higher among patients with sunitinib (11.4 months compared with 5.5 months in the placebo group). Also positive was the objective response rate of 9.3%, with stabilization of the disease in 63% of patients [62]. In this clinical trial, there were 95% of patients with metastases including hepatic metastases in the treatment arm and 94% in the placebo arm (Figure 1). However, this study was stopped early because of the high number of adverse events and deaths in the placebo arm [1, 12].

The mTOR pathway is of great importance since it interferes with the control of cell growth, apoptosis, and protein synthesis. The presence of mTOR pathway abnormalities is acknowledged in NETs, and these abnormalities are also believed to be in part responsible for the development of these tumors. Two mTOR inhibitors have been studied in pancreatic NETs, Temsirolimus, and Everolimus [12].

In a phase II clinical study with Everolimus has demonstrated a response rate of 9.6% and stabilization of the disease in 67.8% of patients [16]. The subsequent phase III study (RADIANT study) confirmed its activity in patients with well and moderately differentiated progressive pancreatic NET with a progression-free survival of 11.1 months and 4.6 months in the placebo arm [63]. In the everolimus arm, 94.8% of the patients had hepatic metastases versus 93.3% of patients in the everolimus plus LAR arm. Treatment was very well tolerated. Most of the adverse effects were grade 1-2 (stomatitis, diarrhea, rash, fatigue, and infections) [1]. 

## 5. Conclusion

The NETs frequently metastasize to the liver, and the presence of liver metastases worsens the prognosis of patients, increasing morbidity and mortality. Different therapeutic options have been developed for the management of patients with NLMs. Surgery resection remains the gold standard, especially in well-differentiated resectable lesions and also improves symptomatic control in selected cases. 

Other liver-directed methods are also advantageous, especially in the presence of predominantly liver disease. In addition, they can be combined with each other and with surgery and systemic therapeutic, allowing a greater range of treatment and increased overall survival and progression-free survival. Those modalities include the RFA, in situations of localized liver damage up to 3 cm of diameter, and HACE, in the case of diffuse liver disease

Systemic therapy is important in controlling symptoms, particularly in carcinoid tumors, with the SSA. For those patients who are not candidates to surgery, SSA can also offer control of disease progression in low grade NET. These agents remain the core of medical therapy for metastatic carcinoid tumors.

In pNETs and particularly in high grade NETs with liver metastases, chemotherapy has a substantial role. 

More recently, the inhibitors of the mTOR pathway and VEGF pathway has been shown very promising in controlling disease progression in pNETs. It is not known their role in high grade tumors and whenever necessary they can be used together with SSA.

There are still some questions that need to be addressed in future studies. For instance, the prevention of tumor progression is one of the main investigating areas. In this area, the role of LAR octreotide (in advanced tumors other than small intestine), lanreotide (investigation is already ongoing in nonfunctioning NET), and pasireotide (SOM230 in NETs from all sites of origin) is being evaluated. The use of everolimus and sunitinib in early stage tumors is another interesting issue, and its role in tumor progression prevention is still to be defined.

Physicians and NET patients are also waiting for guidelines that acknowledge the best treatment approach for patients with grade 3 tumors from any origin and site. The combination of small molecule inhibitors and chemotherapy could be an option. 

The combination of molecular target therapy and chemotherapy, namely, SSA plus chemotherapy, temozolamide and everolimus or temozolamide, and sunitinib in pNET patients should also be addressed in clinical trials.
